# Facilitating transpancreatic biliary sphincterotomy with a rotatable sphincterotome: a case of improved biliary cannulation

**DOI:** 10.1055/a-2515-3951

**Published:** 2025-02-03

**Authors:** Tatsunori Satoh, Kodai Baba, Shinya Kawaguchi, Haruna Takahashi, Shinya Endo, Naofumi Shirane, Kazuya Ohno

**Affiliations:** 1 Department of Gastroenterology, Shizuoka General Hospital, Shizuoka, Japan

To perform endoscopic retrograde cholangiopancreatography (ERCP), cannulation of the common bile duct through the major papilla is necessary. However, even experts fail to achieve selective biliary cannulation in 5% to 20% of cases 1 . When standard cannulation techniques fail, precut incision methods such as needle-knife sphincterotomy and transpancreatic biliary sphincterotomy (TPBS) can be employed. However, these precut techniques also have failure rates of 10% to 40% 2 . Needle-knife sphincterotomy allows for flexible incision lines but is technically challenging, while TPBS is easier but limited by a fixed incision line and difficulty aligning with the bile duct axis. The new rotatable sphincterotome may overcome these limitations, making TPBS easier and more efficient. We herein report a case of successful selective biliary cannulation via TPBS using a novel sphincterotome (ENGETSU; Kaneka Medical, Osaka, Japan).

A 67-year-old woman was admitted to our hospital with obstructive jaundice caused by cholangiocarcinoma ( Fig. 1 ). ERCP was performed using a lateral-viewing endoscope (TJF 290V; Olympus Marketing, Tokyo, Japan), with uneventful insertion and visualization of the papilla. Biliary cannulation attempts using wire-guided and double-guidewire techniques were unsuccessful. We then performed TPBS using the new rotatable sphincterotome for the first time ( Video 1 ). By rotating the sphincterotome, the blade could be directed in any desired orientation ( Fig. 2 **a–c** ). TPBS was carried out with careful alignment to the expected direction of the bile duct axis ( Fig. 2 **d** ). After TPBS, bile duct cannulation was successfully achieved, and the planned procedures (biopsy and stent placement) were completed without complications.

**Fig. 1:**
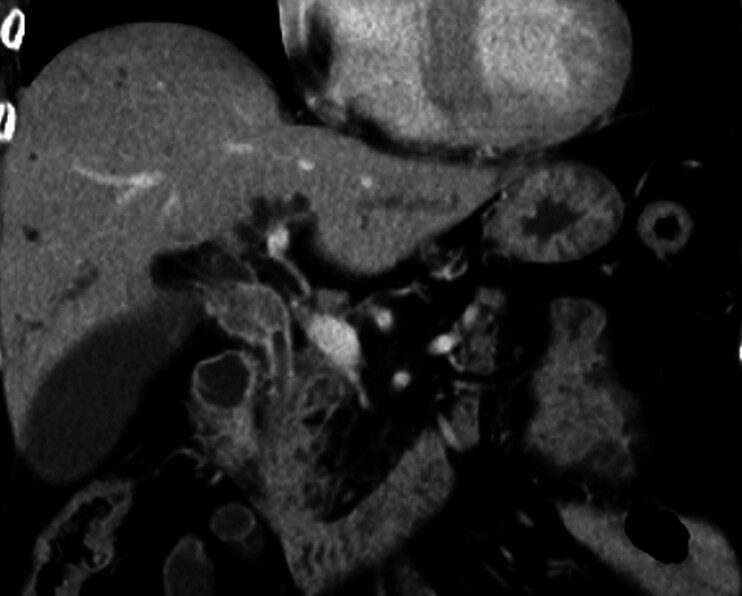
Computed tomography at the time of diagnosis. A mass was identified in the common bile duct, with dilation of the bile duct upstream from the mass.

**Fig. 2:**
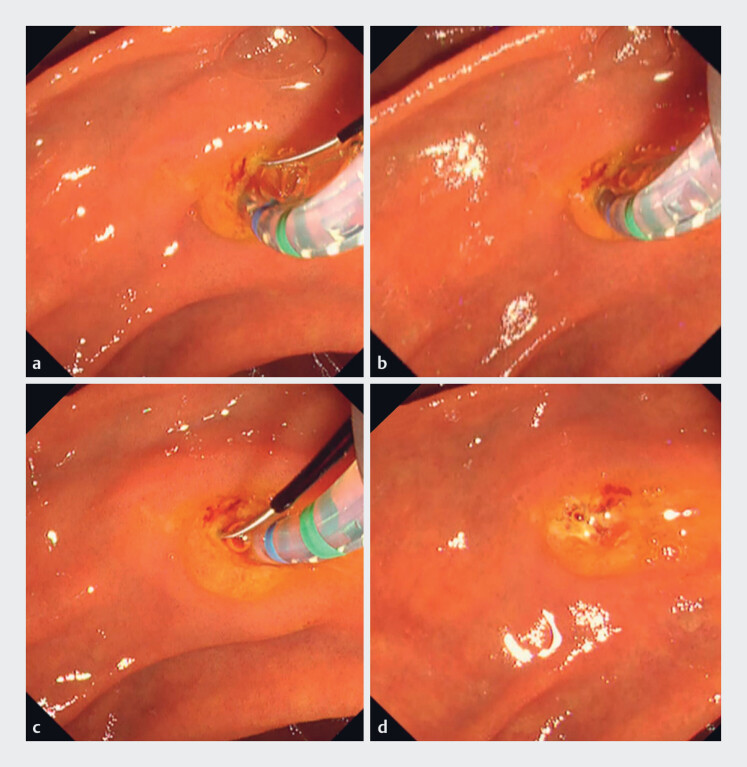
Transpancreatic biliary sphincterotomy (TPBS) using the new rotatable sphincterotome. **a** Neutral position during initial insertion. **b** Position of the sphincterotome after clockwise rotation. **c** Position of the sphincterotome after counterclockwise rotation. **d** Appearance of the papilla after TPBS.

Transpancreatic biliary sphincterotomy using a new rotatable sphincterotome in a case of difficult biliary cannulation.Video 1

This novel sphincterotome enabled effective and safe TPBS in cases where biliary cannulation was difficult.

Endoscopy_UCTN_Code_TTT_1AR_2AC

## Notes

Endoscopy E-Videos https://eref.thieme.de/e-videos
*E-Videos* is an open access online section of the journal *Endoscopy* , reporting on interesting cases and new techniques in gastroenterological endoscopy. All papers include a high-quality video and are published with a Creative Commons CC-BY license. Endoscopy E-Videos qualify for HINARI discounts and waivers and eligibility is automatically checked during the submission process. We grant 100% waivers to articles whose corresponding authors are based in Group A countries and 50% waivers to those who are based in Group B countries as classified by Research4Life (see: https://www.research4life.org/access/eligibility/ ). This section has its own submission website at https://mc.manuscriptcentral.com/e-videos .
